# HAGR-D: A Novel Approach for Gesture Recognition with Depth Maps

**DOI:** 10.3390/s151128646

**Published:** 2015-11-12

**Authors:** Diego G. Santos, Bruno J. T. Fernandes, Byron L. D. Bezerra

**Affiliations:** Escola Politécnica de Pernambuco, Universidade de Pernambuco, R. Benfica, 455-Madalena, Recife-PE 50720-001, Brazil; E-Mails: dgs2@ecomp.poli.br (D.G.S.); byronleite@ecomp.poli.br (B.L.D.B.)

**Keywords:** HCI, dynamic gesture, HMM, DTW, CIPBR

## Abstract

The hand is an important part of the body used to express information through gestures, and its movements can be used in dynamic gesture recognition systems based on computer vision with practical applications, such as medical, games and sign language. Although depth sensors have led to great progress in gesture recognition, hand gesture recognition still is an open problem because of its complexity, which is due to the large number of small articulations in a hand. This paper proposes a novel approach for hand gesture recognition with depth maps generated by the Microsoft Kinect Sensor (Microsoft, Redmond, WA, USA) using a variation of the CIPBR (convex invariant position based on RANSAC) algorithm and a hybrid classifier composed of dynamic time warping (DTW) and Hidden Markov models (HMM), called the hybrid approach for gesture recognition with depth maps (HAGR-D). The experiments show that the proposed model overcomes other algorithms presented in the literature in hand gesture recognition tasks, achieving a classification rate of 97.49% in the MSRGesture3D dataset and 98.43% in the RPPDI dynamic gesture dataset.

## 1. Introduction

Gestures and hand postures have been used for a long time as a way to express feelings and to communicate information between people. A gesture can represent a simple action, such as to allow people to cross a street, or complex body expressions belonging to a specific population language. Sign language uses both hand and body postures instead of sound patterns to establish communication. It is a very important type of language due to the fact that nine million people present some kind of hearing or speaking loss [1], while most people do not speak sign language, as most of the hearing impaired are illiterate in their local language. Gesture recognition can work on this problematic by creating a bridge between the languages, recognizing a given gesture and translating it into words in real time [2].

There are three types of gesture recognition systems: based on devices attached to the body [3], based on gesture tracking [4] and based on computer vision techniques [5]. The first category uses sensors, such as wearable devices with accelerometers and markers, to capture a gesture and its corresponding movement. However, this invasive technology limits the normal execution of the gesture.

Gesture recognition systems based on tracking aim to follow the gesture trail, drawing a path through its execution using a marker. The limitation comes from the fact that these systems use only the traveled path, and they do not perform well with complex and very detailed movements, such as the ones involving different hand postures.

The last category, systems based on computer vision techniques, uses a camera device to capture the gesture and extract features, such as the speed, direction and intensity of a given gesture. Due to variations in gesture execution, people executing them and the environment, the accuracy of such systems can be degraded in specific scenarios, like reduced illumination, very fast movement or interference from other people [6,7]. However, it is the least invasive category, allowing a more natural iteration between the user and the system without impairing the gesture execution. Usually, some steps between the image capture and the classification output are followed by these systems: image segmentation, feature extraction and pattern classification [8]. In the first step, the image background is removed, and only the body parts relevant to the gesture recognition are kept [9]. In this scenario, the Microsoft Kinect [10] appeared as an interesting solution to gesture recognition, presenting an important contribution in image segmentation for body detection. In the work of Tara *et al.* [11], the Microsoft Kinect is used to capture depth maps and to recognize static gestures. The depth maps are used to detect the hand through the definition of a distance threshold in which the hand is located. Lee *et al.* [12] proposed an approach with k-means and the convexity hull to find the fingers and provide a more accurate analysis of the gesture. The main proposition of Palacios *et al.* [13] consists of a segmentation algorithm where the user does not need to execute the gesture in the front of the body and near the depth sensor.

In the second step of gesture recognition systems based on computer vision techniques, descriptors are extracted in order to computationally represent the gesture pattern [14]. Thus, the images are reduced to feature vectors by using mathematical models [15]. Oreifej and Liu [16] proposed a technique called Histogram of Oriented 4D Normals (Hon4d) that uses a 4D histogram approach for feature extraction, while Yang [17] proposed an algorithm for 2D and 3D spaces that extracts some features from the executed gestures: the location of the left hand with respect to the signer’s face in 3D space; the angle from the face to the left hand; the position of the left hand with respect to the shoulder center; the occlusion of both hands. Doliotis *et al.* [18] proposed a feature extraction method using images generated by a Microsoft Kinect, retrieving a 3D pose orientation and full hand configuration parameters.

It is also important to note that there is a common issue between many feature extraction methods in gesture recognition: the curse of dimensionality [19]. Some approaches have been proposed to solve this problem, like the reduction of the feature vectors [20,21] by selecting a smaller set of features that adequately keeps the original representation in order to distinguish the different gestures. Probabilistic models analyze the correlation between the features allowing their selection, like Principal Component Analysis (PCA) or Independent Component Analysis (ICA) [22]. Moreover, optimization function techniques can also be used to reduce the feature vectors, aiming to minimize the model error rate, such as swarm methods [23], which are designed to optimize high dimensionality functions.

In the last step of gesture recognition of systems based on computer vision techniques, a classifier is trained using the extracted descriptors in order to recognize the gestures [24]. Barros *et al.* [5,25] presented a gesture recognition system that achieved higher classification rates in comparison to other methods using dynamic time warping (DTW) [26] and hidden Markov model (HMM) [27] classifiers. Kim *et al.* [28] also used DTW as a classifier to recognize gestures captured by a depth sensor. Godoy *et al.* [29] proposed a gesture recognition method trained on a few samples with HMM, achieving high classification rates. Neverova *et al.* [30] proposed a framework based on a multi-scale and multi-modal deep learning architecture, which is able to detect, locate and recognize a gesture. To complete this task, they used information obtained from different data channels of a depth image, decomposing the gesture into multiple temporal and spatial scales. Wu *et al.* [31] proposed a multilayered gesture recognition system, dividing the recognition phase into three layers: the first layer for fast distinguishing types of gestures based on PCA; the second layer is a particle-based descriptor to extract and identify dynamic information from gestures in each frame using DTW with adaptive weights; and finally, the static hand shapes are recognized in the third layer. Their study achieved significant results in a large dataset with 50,000 gestures [32].

In this paper, we propose a novel approach for dynamic gesture recognition with depth maps, called the hybrid approach for gesture recognition with depth maps (HAGR-D). HAGR-D uses a version of CIPBR (convex invariant position based on RANSAC) algorithm [33] for feature extraction, a combination of the binary particle swarm optimization [34] and a selector algorithm to make the feature selection and a hybridization between DTW and HMM classifiers for recognition. DTW is used to find the most probable gestures, while HMM refines DTW output.

This paper is organized as follows. Section 2 describes the proposed model. In Section 3, experiments with gesture images captured by the Microsoft Kinect are shown. Finally, in Section 4, we present some concluding remarks.

## 2. Hybrid Approach for Gesture Recognition with a Depth Map

**Figure 1:**
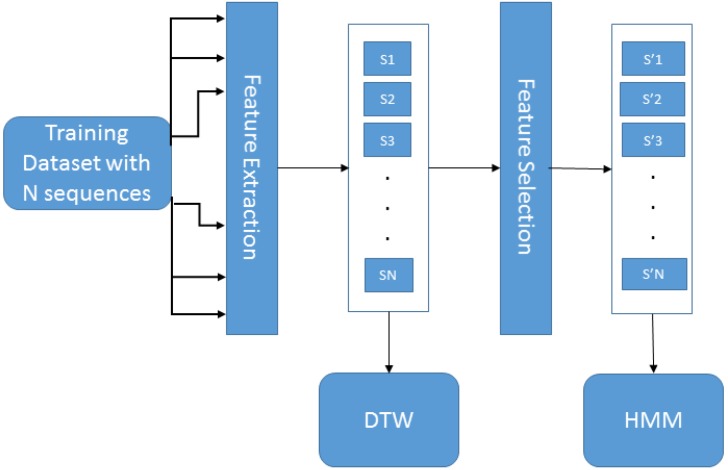
HAGR-D training architecture.

HAGR-D is an approach for gesture recognition that involves a method for feature extraction, a method for feature vector reduction and a hybrid classifier. Figure 1 presents the training architecture for the HAGR-D system, which starts with feature extraction using a variation of the CIPBR algorithm for depth maps. These vectors are used to train the DTW classifier. The feature selection method uses a combination between binary particle swarm optimization [34] and a selector algorithm [25] to reduce the feature vector that is used by the HMM to refine the DTW classification result. We present the depth CIPBR algorithm in Section 2.1, the feature selection method in Section 2.2 and a description of DTW and HMM hybridization in Section 2.3. Table 1 presents the notations and definitions used to describe the HAGR-D.

**Table 1:** Notations and definitions used to describe the HAGR-D.

Symbol	Description
C	Hand contour center of mass
P	The hand contour highest point
$\bar{PC}$	The line segment between points *P* and *C*
Θ	Maximum circumcircle
Ψ	Convex hull points of the hand contour
$p_{n}$	Contour point of the convex hull Ψ
*ω*	Point of the Ψ set
$\bar{\omega C}$	Line segment calculated between the points *ω* and *C*
*D*	Set of distances
*Q*	Intersection between $\bar{\omega C}$ and Θ
*d*	Distance from the *D* set
$d^{\prime }$	distance *d* normalized
*A*	Set of angles
*a*	Angle from the *A* set
$a^{\prime }$	Angle *a* normalized
*F*	Final feature vector returned by depth CIPBR
*X*	Set of position in a search dimension
$x_{n}$	Position in a search dimension
*V*	Set of velocities in a search dimension
$v_{n}$	Velocity in a search dimension
$p_{Best}$	Best position found by a particle
$g_{Best}$	Best position found by the swarm
*S*	Feature vector
$S^{\prime }$	Reduced feature vector
*k*	Number of gestures candidates returned by DTW in classification time
CM	Cost matrix generated for DTW to compare two patterns
*i*	Size of *A* pattern feature vector and position in a cost matrix row
*j*	Size of *B* pattern feature vector and position in a cost matrix column

### 2.1. Depth CIPBR

**Figure 2:**
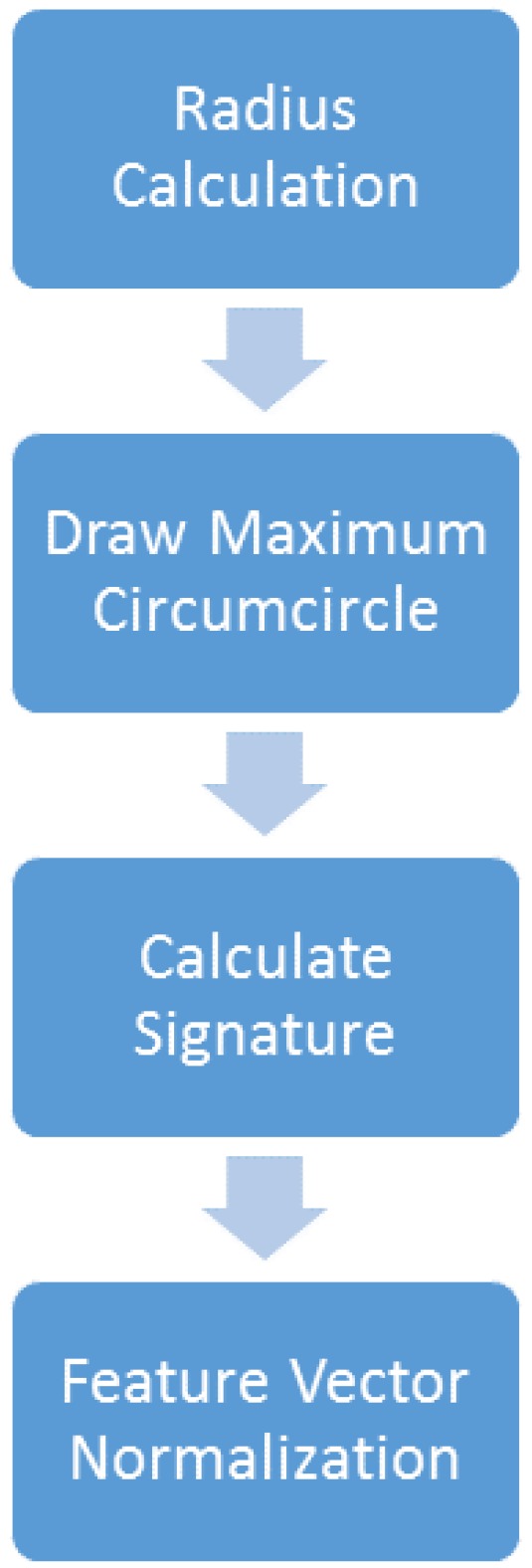
Depth CIPBR architecture.

The depth CIPBR algorithm is an approach composed of a sequence of tasks to reduce a depth map of a hand posture into two signature sets proposed by Keogh *et al.* [35]. To complete these tasks, there are four modules connected in cascade, as presented in Figure 2.

**Figure 3:**
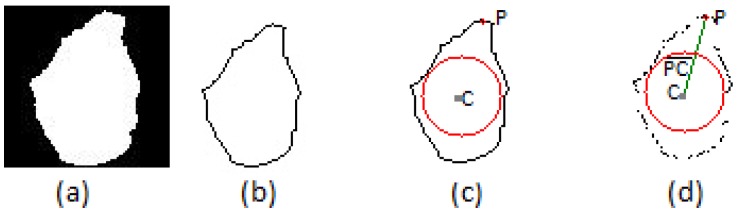
(**a**) The hand segmented using the Microsoft Kinect; (**b**) the hand posture contour; (**c**) the mass center point drawn in the hand posture contour (dark gray point); (**d**) the convex hull points with the maximum circumcircle Θ (red circle), the center mass point(dark gray point), the highest point (red point) and the segment of line $\bar{PC}$ (green line).

The first module, “radius calculation”, uses a hand posture image that is segmented from the depth map generated by the Microsoft Kinect; Figure 3a. The hand posture contour is extracted from this image, generating the contour (Figure 3b), and the center of mass (*C*) of the hand posture is calculated from the image contour using the center moments [36]. Then, the point that has the lower Y coordinate is found, *P*; Figure 3c. Finally, this module calculates the distance between the center of mass and point *P*. Figure 3d presents an output example of the “radius calculation” module. The dark gray point is the center of mass of the contour given by *C*; the red point is the highest point of the contour given by *P*; and the line connecting these points is given by $\bar{PC}$.

The second module of Depth CIPBR, “draw maximum circumcircle”, uses the line segment $\bar{PC}$ as radius to draw a circle inside the hand contour. If this circle exceeds the hand contour boundary, a triangle is calculated using the three most distant contour points from the point *C*, two of them being on opposite sides of the contour. The biggest circle inside this triangle is the maximum circumcircle Θ of the contour with the center at point *C*.

The third module of Depth CIPBR, “calculate signatures”, receives the maximum circumcircle Θ and points *P* and *C* as input. The hand contour points are substantially reduced using Andrew’s monotone chain convex hull algorithm [37].

Andrew’s algorithm outputs a set $\Psi =\{p_{1},p_{2},\ldots ,p_{n}\}$ from convex hull points, which is used to generate two signature sets. The first signature set is composed of distances (D) calculated as follows:

- For each point ω∈Ψ, the length of the line segment $\bar{\omega C}$ is calculated based on the Euclidean distance from *ω* to the point *C*;
- Then, this length is subtracted from the circumcircle radius, in order to obtain the $\bar{\omega Q}$ length, where point Qis the intersection between segment $\bar{\omega C}$ and Θ.

Therefore, the first signature set is composed of each distance $D_{\bar{\omega Q}}$, ∀ω∈Ψ, calculated using the following Equation (1), where:

- *C* is the center of mass of the hand posture contour;
- $\omega_{x},C_{x}$ are the *x* coordinates for points ω and C, respectively;
- $\omega_{y},C_{y}$ are the *y* coordinates for points ω and C, respectively;
- radius is the radius of Θ calculated by the “draw maximum circumcircle” module.

The second signature set consists of a vector of angles obtained by calculating the angle between a line composed of each point w∈Ψ of the convex hull hand shape point and the line segment $\bar{PC}$. Both signature sets are obtained in a clockwise direction, always starting with point *P*.

Finally, in the last module, “feature vector normalization”, the signature sets are normalized. The first signature set is normalized dividing each distance by the radius calculated in the “draw maximum circumcircle” module. The normalized distance vector is represented by $D^{\prime }=\left\{d_{1}^{\prime },d_{2}^{\prime },\ldots ,d_{n}^{\prime }\right\}$:

(1) $$d_{i}^{\prime }=\frac{d_{i}}{radius}$$

The set of angles is normalized by dividing each angle by $360^{\circ }$:

(2) $$a_{i}^{\prime }=\frac{a_{i}}{360^{\circ }}$$

Angle and distance sets are concatenated in the following order: angles first and distances at the end of the signature vector. Therefore, the final feature vector is $F=\{\;a_{1}^{\prime },\;a_{2}^{\prime },\;\ldots ,\;a_{n}^{\prime },\;d_{1}^{\prime },\;d_{2}^{\prime },\;\ldots ,\;d_{n}^{\prime }\}$.

### 2.2. Feature Selection Method

Some classifiers used for gesture recognition are more sensitive to the curse of dimensionality [19], such as the HMM [38,39]. In order to overcome this obstacle, the feature selection method finds the smallest size possible for the feature vector and assigns the same size for the feature vectors of all gestures. This is also an important task, since many classifiers use inputs with the same predefined size.

In this study, binary particle swarm optimization (BPSO) [34] finds the target size of the reduced feature vector, while the selector algorithm is used to resize the feature vectors. The objective that BPSO seeks to optimize is the minimum distance between the particle composed of zeros and ones and the gestures sequences. The number of ones in the particle denotes the size of the new feature vector.

The next subsections explain in detail how these algorithms work.

#### 2.2.1. Particle Swarm Optimization

Particle swarm optimization [40] solves an optimization problem with a swarm of simple computational elements, called particles, exploring a solution space to find an optimal solution. The position from each particle represents a candidate solution in *n*-dimensional search space (D) defined as $X=\{x_{1},x_{2},x_{3},\ldots ,x_{n}\}$, where each $x_{n}$ is a position in the *n*-dimension, and the particle velocity is represented by $V=\{v_{1},v_{2},v_{3},\ldots ,v_{n}\}$.

The fitness function evaluates how well each particle presents itself in each iteration. When a particle moves and its new position has a better fitness value than the previous one, this value is saved in a variable called $p_{best}$. To guide the swarm to the best solution, the position, where a single particle found the best solution until the current execution, is stored in a variable called $g_{best}$. Therefore, to update the particle velocity and position, the following equations are used:

(3) $$v_{i}\left(t+1\right)=\kappa v_{i}\left(t\right)+c_{1}r_{1}\left[p_{i,best}-x_{i}\left(t\right)\right]+c_{2}r_{2}\left[g_{best}-x_{i}\left(t\right)\right]$$

(4) $$x_{i}\left(t+1\right)=x_{i}\left(t\right)+v_{i}\left(t+1\right)$$

where i=(1,2,3,…,N), *N* is the size of the swarm, $c_{1}$ represents the private experience or “cognitive experience” and $c_{2}$ represents the ”social experience” interaction, usually used with a value of 2.05 [40]. Variables $r_{1}$ and $r_{2}$ are random numbers between zero and one and represent how much $p_{best}$ and $g_{best}$ will influence the particle movement. The inertia factor *κ* is used to control the balance of the search algorithm between exploration and exploitation. The $x_{i}$ represents the particle position in the *i*-th dimension. The recursive algorithm runs until the maximum number of iterations is reached.

#### 2.2.2. Binary PSO

The binary PSO is a variation of the traditional PSO in discrete spaces. The major difference between this algorithm and its canonical version is the interpretation of velocity and position. In the binary version, the particle’s position and velocity are represented by zeros and ones only. This change requires a reformulation in how velocity is calculated, according to the following equation:

$$\begin{array}{c} If\;rand\;<\;\frac{1}{1+e^{-v_{i}\left(t+1\right)}}\;then\; \end{array}$$

(5) $$\begin{array}{c} x_{i}\left(t+1\right)=1;\;else\;x_{i}\left(t+1\right)=0 \end{array}$$

where *rand* is a random number between zero and one.

Finally, to binarize all of the feature vectors, a threshold calculated through the mean of all of the feature vectors is used. BPSO calculates a distance from each $x_{ij}$ binary particle’s position to the same *j* position in all binary vectors for the same gesture. After each iteration, all distances are added up to generate the fitness function output. Particles are improved as soon as the fitness values become smaller in comparison with the fitness obtained by the previous iteration. The particle fitness function is:

(6) $$fitness_{i}=\sum_{j=1}^{m}\left[\sqrt{\sum_{k=1}^{n}{\left(x_{ik}-F_{jk}\right)}^{2}}\right]$$

where $\left(x_{i1},\;x_{i2},\;\ldots ,\;x_{in}\right)$ is the particle’s *i*-th position and $\left(F_{j1},\;F_{j2},\;\ldots ,\;F_{jn}\right)$ is the *j*-th features in all vectors.

#### 2.2.3. Selector Algorithm

BPSO chooses the target size for the reduced feature vector $S^{\prime }$. Then, the selector algorithm [25] reduces the CIPBR feature vector *S* to $S^{\prime }$, producing the final vectors of the proposed approach. In this process, some rules must be respected. First, if any vector has fewer points than the target size of $S^{\prime }$, zeros are added to the feature vector until it matches the desired length. Second, feature vectors larger than the target size of $S^{\prime }$ are redefined using a selection algorithm. This algorithm consists of calculating a window *W* through the division of the current vector length by the target size of $S^{\prime }$. The current vector *S* is parsed, and each value in the *W* position is included in the new feature vector. If the new output vector $S^{\prime }$ is even smaller than the desired length, the remaining positions are randomly visited in *S* and used to compose the new output vector $S^{\prime }$ until the desired length is reached.

### 2.3. DTW and HMM Hybridization

In order to classify the depth CIPBR feature vectors, a hybridization between two classifiers that generated good results in the literature of dynamic gesture classification is proposed: DTW and HMM [5,28,29].

DTW gives the distance between two patterns that represents the degree of similarity between them using a cost matrix (CM). Given two patterns $A=\{a_{1},a_{2},\ldots ,a_{N}\}$ and $B=\{b_{1},b_{2},\ldots ,b_{N}\}$, the cost matrix cell $CM_{i,j}$ is the distance calculated between the element $x_{i}$ and $y_{j}$. The similarity degree will be the sum of the lowest cost path in the matrix, which starts at $CM_{1,1}$ and finishes at $CM_{i,j}$.

DTW works well in classifying grouped patterns, but it is not very sensitive to very close patterns and might commit some mistakes. We observed that in most DTW misclassifications, the right output was near the compared gesture of the training dataset. Thus, we propose to refine DTW output with HMM to reduce the number of mistakes.

A hidden Markov model (HMM) is a statistical Markov model in which the system being modeled is assumed to be a Markov process with unobserved (hidden) states. A simple way of observing an HMM is imagining it as a finite automaton deterministic with two alphabets, that is in every state, it will tell the likelihood of a hand posture change to another depending on the executed gesture. The HMM has the quality of being fast in training and running, but it can be very fragile in assertiveness, because all training depends on how likely matrices are initiated. Thus, with a higher initial matrix, or amount of the class involved, the HMM will loses some of its power.

DTW has no training phase, but retains a certain number of examples, so it can make the comparison between the kept examples and some input pattern, returning the class belonging to the closest example to the input pattern. Because of this proximity, DTW often confuses a class of a gesture with its closest neighboring class. The canonical DTW [26], being the only classifier, faces the problem of proximity between classes of feature vectors generated by the CIPBR algorithm. Because of this proximity between classes, DTW has a greater tendency to return a foreign class present in the training set among the supposedly correct class examples.

To work around this problem, DTW is used in the hybrid classifier in order to return no longer a class, but the closest sequences to the input pattern belonging to training set. Thus, the correct class is more likely to be among the returned sequences. At this stage, the trained HMM has a higher probability of returning the correct class, avoiding the transition matrices outliers, since HMM only needs to classify between the sequences returned by DTW.

**Figure 4:**
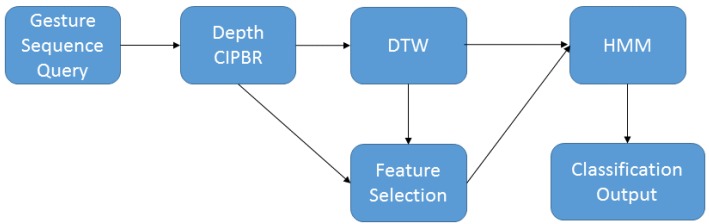
HAGR-D classification architecture.

Figure 4 presents the sequence performed by the HAGR-D for gesture recognition. First, DTW classifies the gesture using the CIPBR algorithm for feature extraction and returns the *k* nearest gestures of the input sequence as candidates. The input sequence is then resized by the selector algorithm using the best size found by the BPSO in training time, and HMM decides the classification of the input sequence between the *k* candidate gestures returned by DTW.

Algorithm 1 presents a pseudo-code for DTW and HMM hybridization. The model starts receiving a set with images from a gesture at Line 1. The Depth CIPBR algorithm extracts the features of the hand postures in Line 2, and DTW classifies this feature vector and outputs another vector with the *k* nearest gestures from the training dataset, Line 3. Then, the input gesture and the outputs of DTW are resized by the selector algorithm using the size that the BPSO decided in the training time, Line 6. Finally, the HMM classifies each resized vector, and the hybrid classifier outputs the most incident class as returned by the HMM, Lines 7–9.

**Algorithm 1** Pseudo-Code from DTW and HMM Hybridization.1: Images ← gesture images of Image2: features ← depthCIPBR.extracts(Images)3: dtwOutputs ← dtw.classify (features)4: hmmOutpts5: **for** each gesture in dtwOutputs **do**6:     resizedSequence ← selectorAlgorithm.resize(gesture, size)7:     hmmOutpts ← hmm.classify(resizedSequence)8: classification ← mostIncidence(hmmOutputs)9: **return** classification

## 3. Experimental Results

In order to evaluate the HAGR-D, two experiments are performed with public benchmarks: the MSRGesture3D dataset [41] and the RPPDI dynamic gesture dataset [5]. The next subsections explain these experiments in detail.

### 3.1. MSRGesture3D

The MSRGesture3D is a dynamic hand gesture dataset captured by the Kinect RGB-D camera. There are 12 dynamic hand gestures defined by American Sign Language (ASL) in MSRGesture3D, and each dynamic gesture was performed two or three times by each one of 10 subjects. The gestures presented in the dataset are: “bathroom”, “blue”, “finish”, “green”, “hungry”, “milk”, “past”, “pig”, “store”, “where”, “J” and “Z’.’ The dataset contains only depth data images and is considered challenging mainly because of self-occlusion issues. We used the leave-one-subject-out cross-validation to evaluate the dataset as proposed in [41].

The following parameters are used for training the HAGR-D:

- DTW: k=5;
- BPSO:
  - 15 particles;
  - 20 dimensions;
  - 30 simulations;
  - 200 iterations;
  - c1=c2=2.05;
  - inertia factor of w=0.9→0.4;
  - r1=r2=rand(0...1);
- HMM:
  - three states;
  - 100 iterations.

To find the initial states of HMM, we use a k-means clustering [42] technique, avoiding the random initial matrices, while the BPSO uses few dimensions in its particles to guarantee small final vectors. The works in [43,44] use similar approaches to determine the final size of the vectors in their studies.

**Figure 5:**
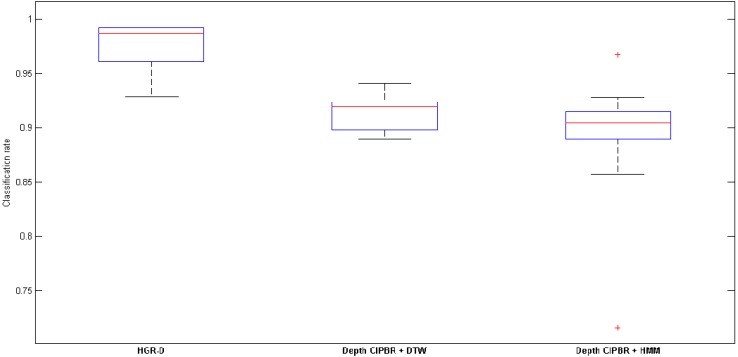
Comparison between HAGR-D, depth CIPBR with DTW and depth CIPBR with HMM for hand gesture recognition.

We compared the HAGR-D with a model using the same feature extraction approach, but with DTW or HMM alone as classifiers, “called depth CIPBR + DTW” and “depth CIPBR + HMM”. Therefore, we are able to evaluate the performance of the proposed model with DTW before HMM and solely with HMM. The inputs for DTW are the raw sequences generated by depth CIPBR, and the inputs for the HMM are the output sequences of the feature selection method. Figure 5 presents the boxplot for each method. It is easy to see that the hybridization between DTW and HMM significantly improved the proposed model. Furthermore, in several iterations, the HAGR-D classification rate was very close to 100%, and only two sequences were misclassified, corresponding to the gesture “green” being classified as “store” and the gesture “blue” being classified as “where”. Another point to be made is that outliers presented using solely HMM as a classifier no longer exist with HAGR-D. The size of the boxplot generated by the results of each classifier also elucidate a low variation between the results of each classifier, providing a certainty about the consistency of the results.

**Figure 6:**
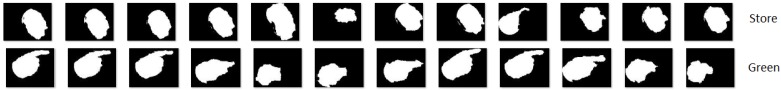
Examples of sequences of the gestures store and green.

Table 2 and Table 3 present the confusion matrices of HAGR-D and “depth CIPBR + DTW”, respectively, applied to the MSRGesture3D dataset. As can be seen, the hybridization of the “depth CIPBR + DTW” with HMM generating HAGR-D improved the classification rates, and most of the HAGR-D mistakes happened between the gestures “green” and “store”, with 7% of the “green” being classified as “store”. Figure 6 shows an example of each of these gesture sequences and how close their hand postures are.

**Figure 7:**
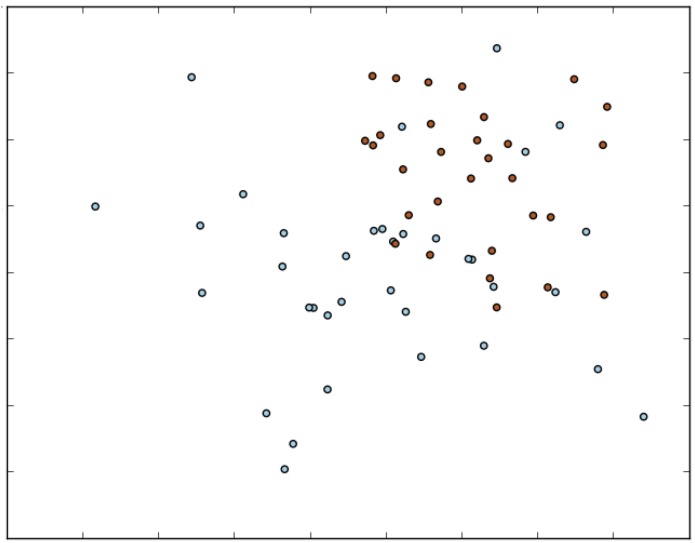
Example of two generated classes by the CIPBR algorithm.

Figure 7 shows a representation of the two gesture vectors most confused by HAGR-D and how close they are. To represent this gesture in a 2D space, they are normalized in size using the “selector algorithm” with only two features as the final length. It is easy to see that sometimes, a few examples cross the division between classes, making classification more difficult.

**Table 2:** Confusion matrix of the MSRGesture3D database results classified by HAGR-D.

	Z	J	Milk	Where	Store	Pig	Past	Green	Finish	Bathroom	Hungry	Blue
Z	100%	-	-	-	-	-	-	-	-	-	-	-
J	-	95%	-	-	-	-	-	-	-	-	-	5%
Milk	-	-	100%	-	-	-	-	-	-	-	-	-
Where	-	2%	-	94%	-	-	4%	-	-	-	-	-
Store	-	5%	-	-	95%	-	-	-	-	-	-	-
Pig	-	-	-	-	-	100%	-	-	-	-	-	-
Past	-	-	-	-	3%	-	92%	-	4%	-	1%	-
Green	-	-	-	-	7%	-	-	93%	-	-	-	-
Finish	-	-	-	-	-	-	-	-	100%	-	-	-
Bathroom	-	-	-	-	-	-	-	-	-	100%	-	-
Hungry	-	-	-	-	-	-	-	-	-	-	100%	-
Blue	-	-	-	2%	-	-	-	-	-	-	-	98%

**Table 3:** Confusion matrix of the MSRGesture3D database results classified by the CIPBR + DTW combination.

	Z	J	Milk	Where	Store	Pig	Past	Green	Finish	Bathroom	Hungry	Blue
Z	92%	-	-	4%	-	-	-	-	2%	-	-	2%
J	-	90%	-	-	-	-	4%	1%	-	-	-	5%
Milk	-	-	98%	2%	-	-	-	-	-	-	-	-
Where	-	4%	-	91%	-	-	5%	-	-	-	-	-
Store	-	10%	-	-	90%	-	-	-	-	-	-	-
Pig	-	-	-	-	-	93%	3%	-	-	-	-	-
Past	-	-	-	-	3%	-	89%	-	7%	1%	-	-
Green	-	-	-	-	14%	-	-	82%	4%	-	-	-
Finish	-	-	-	-	-	-	-	-	100%	-	-	-
Bathroom	2%	-	-	-	3%	-	5%	-	-	90%	-	-
Hungry	-	-	-	-	-	-	-	-	-	-	100%	-
Blue	-	-	2%	2%	-	-	-	-	-	-	-	96%

Finally, Table 4 presents the results obtained for the MSGesture3D dataset using the leave-one-subject-out cross-validation as a testing procedure in comparison with other methods in the literature. As presented, HAGR-D achieved the best classification result of 97.49%.

**Table 4:** Comparison between the results for MSRGesture3D with the leave-one-subject-out cross-validation as the classification process.

Method	Classification Rate (%)
HAGR-D	97.49
Depth CIPBR + DTW	91.53
Depth CIPBR + HMM	88.98
Actionlet [45]	95.29
HON4D + $D_{disc}$ [16]	92.45
HON4D [16]	87.29
ROP, Wang *et al.* [41]	88.50
Depth motion maps, Yang *et al.* [46]	89.20
Kurakin *et al.* [47]	87.70
Klaser *et al.* [48]	85.23

Venkateswara *et al.* [49] use the same dataset in their study, but modifying the experiment using five subjects for training and five for testing their methods, achieving 94.6% as their best result, which is still above ours.

### 3.2. RPPDI Dynamic Gesture Dataset

**Figure 8:**
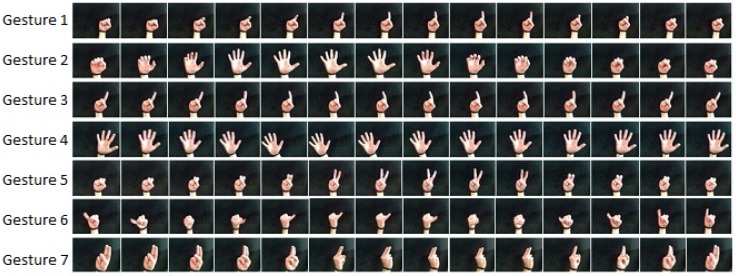
Example for each of the gestures performed on the RPPDI dynamic gesture dataset.

The RPPDI dynamic gesture dataset is a set of images of seven dynamic hand gestures performed in front of a smartphone camera. Figure 8 illustrates one sequence example for each gesture in the dataset. Each gesture is performed several times, and Table 5 presents the number of sequences in each gesture. We used the same test configuration as proposed by Barros *et al.* [5,25] with 2/3 of the dataset for training the model and 1/3 for testing.

**Table 5:** Number of sequences captured by each gesture in the RPPDI dynamic dataset.

Gesture	Number of Sequences
Gesture 1	24
Gesture 2	24
Gesture 3	31
Gesture 4	18
Gesture 5	26
Gesture 6	33
Gesture 7	32

The experiments in the RPPDI dataset used the same configuration for BPSO and HMM as presented in Section 3.1, and the results are compared to Barros *et al.*’s previous works. The CIPBR uses the Otsu threshold [50] as a binarization method in the first module to segment hand posture. Table 6 presents the results obtained, and Table 7 presents the confusion matrix of the HAGR-D system. The proposed method committed a few mistakes, misclassifying only one gesture, while achieving 100% accuracy in some iterations.

**Table 6:** Comparison between the results in RPPDI dynamic gesture dataset.

Method	Classification Rate (%)
HAGR-D	98.43
Speed Up Robust Features (SURF) + HMM [25]	75.00
Local Contour Sequence (LCS) + HMM [25]	77.00
Convex SURF (CSURF) + HMM [25]	91.00
Convex LCS (CLCS) + HMM [25]	91.00
SURF + DTW [25]	38.00
LCS + DTW [25]	78.00
CSURF + DTW [25]	93.00
CLCS + DTW [25]	97.00

**Table 7:** Confusion matrix of RPPDI dynamic gesture database classified by the HAGR-D system.

Gesture	1	2	3	4	5	6	7
1	100%	-	-	-	-	-	-
2	-	100%	-	-	-	-	-
3	-	-	89%	-	-	11%	-
4	-	-	-	100%	-	-	-
5	-	-	-	-	100%	-	-
6	-	-	-	-	-	100%	-
7	-	-	-	-	-	-	100%

### 3.3. Remarks

The proposed approach, HAGR-D, achieved the best results in two different datasets due to the combination of depth CIBPR for feature extraction and the hybrid classifier with DTW and HMM. The classifiers compensated the failures of each other by reducing misclassifications between different gestures with DTW and refining the classification output through validation of the most similar sequences using the HMM. The hybrid classifier improved the results in comparison with DTW and HMM applied individually.

One of the limitations of HAGR-D is the definition of the number of sequences returned by DTW, *k*. In this study, such a parameter was empirically defined. Another limitation is the computation cost of DTW that might impair real-time application of the proposed model.

Another point to be made is that the few mistakes committed by HAGR-D were due to very similar sequences. Nevertheless, many conditions must be fulfilled in order for HAGR-D to misclassify a given gesture: the number of similar postures, the distance from the hand to the sensor, the speed of gesture execution and occlusion.

## 4. Conclusions and Future Works

Hand gesture recognition for real-life applications is very challenging because of its requirements of robustness, accuracy and efficiency. In this paper, we proposed both a variation of the CIPBR algorithm for depth maps and a hybrid classifier for gesture recognition using DTW and HMM. The proposed approach, HAGR-D, presents better results than the ones in the literature, achieving a classification rate of 97.49% in the MSRGesture3D dataset and 98.43% in the RPPDI dynamic gesture dataset. The application of depth CIPBR for feature extraction showed good results, while the hybridization between the DTW and HMM classifiers significantly improved classification accuracy.

Although the focus of classification in this paper relies on the task of hand gesture recognition, in future research, we intend to extend the application of the HAGR-D to other types of gestures, such as human body movements. Furthermore, the DTW has a high computational cost, which makes the HAGR-D execution slow; however, the FastDTW [51] is a variation of the traditional form of the DTW that promises to exponentially reduce the computational cost, and it will be addressed in our next experiments.
